# Inducible nitric oxide synthase and systemic lupus erythematosus: a systematic review and meta-analysis

**DOI:** 10.1186/s12865-020-0335-7

**Published:** 2020-02-17

**Authors:** Lu Pan, Sirui Yang, Jinghua Wang, Meng Xu, Shaofeng Wang, Huanfa Yi

**Affiliations:** 1 Central Laboratory, The First Hospital of Jilin University, Changchun, China; 2 Department of Pediatric Rheumatology and Allergy, The First Hospital of Jilin University, Changchun, China; 3 The Institute of Epigenetic Medicine, The First Hospital of Jilin University, Changchun, China

## Abstract

**Background:**

There is a growing body of evidences indicating iNOS has involved in the pathogenesis of SLE. However, the role of iNOS in SLE is inconsistency. This systematic review was designed to evaluate the association between iNOS and SLE.

**Results:**

Six studies were included, reporting on a total of 277 patients with SLE. The meta-analysis showed that SLE patients had higher expression of iNOS at mRNA level than control subjects (SMD = 2.671, 95%CI = 0.446–4.897, z = 2.35, *p* = 0.019), and a similar trend was noted at the protein level (SMD = 3.602, 95%CI = 1.144–6.059, z = 2.87, *p* = 0.004) and positive rate of iNOS (OR = 9.515, 95%CI = 1.915–47.281, z = 2.76, *p* = 0.006) were significantly higher in SLE group compared with control group. No significant difference was observed on serum nitrite level between SLE patients and control subjects (SMD = 2.203, 95%CI = -0.386–4.793, z = 1.64, *p* = 0.095). The results did not modify from different sensitivity analysis, representing the robustness of this study. No significant publication bias was detected from Egger’s test.

**Conclusions:**

There was a positive correlation between increasing iNOS and SLE. However, the source of iNOS is unknown. Besides NO pathway, other pathways also should be considered. More prospective random studies are needed in order to certify our results.

## Background

Systemic lupus erythematosus (SLE) is a chronic, autoimmune disease characterized by a variety of clinical symptoms, producing autoantibodies and causing damage to multiple organs [1–3]. However, the pathogenesis of SLE remains unclear. Up to now, T-cell and B-cell abnormalities, impaired apoptotic debris clearance, abnormal cytokines and autoantibody productions have been partially set forth [4]. In recent years, free radical-mediated reactions have been implicated as contributions in a range of autoimmune diseases, including SLE [5, 6].

Nitric oxide (NO) is one of the most important and widely studied free radical molecules. It plays an important role in the regulation of physiological processes, host defense, anti-inflammation under physiologic conditions [7, 8]. Conversely, it serves as a cytotoxic effector molecule or a pathogenic mediator of tissue destruction when over-expressed [9]. Previous study showed NO is an important mediator of apoptosis and a vital regulator of the Th1/Th2 balance in autoimmune diseases [10]. Furthermore, over-expression of NO was parallel with the development of SLE [11, 12]. However, NO is unstable, its potential in SLE pathogenesis lies largely on the extent of its production and generation of O_2_^▪ -^, leading to formation of peroxynitrite (ONOO^−^). ONOO^−^ is one of reactive nitrogen species (RNS), which induced tissue injury via direct oxidant, protein tyrosine nitration and nucleic acid modification [5, 13–15]. In addition, it can penetrate the cellular membranes directly and oxidate protein side-chains which may be related to oxidative cell injury [16, 17]. Alteration of protein can generate neo-epitopes, which may be effective in activating T cells, leading to autoimmune attack. Accumulating evidence showed a positive association between RNS and SLE [14, 18]. Meanwhile, blocking RNS can reduce disease activity [19]. Moreover, it had been showed that NO production, such as nitrate and nitrite, as well as RNS-modified proteins, such as 3-nitrotyrosine (3-NT) were elevated in SLE [20, 21]. Therefore, NO plays an important role in SLE via different ways. Nitric oxide synthase (NOS) is the synthase catalyze L-arginine to form NO.

There are three isoforms of NOS: neuronal NOS (nNOS), endothelial NOS (eNOS), and inducible NOS (iNOS). In contrast with the former two isoforms, iNOS is an inducible and calcium-independent synthase. Studies in murine models have shown that iNOS is induced in response to inflammatory stimuli, and iNOS can produce much higher amounts of NO than the other two isoforms. More and more evidence showed a positive correlation between increasing iNOS activity and progression of SLE. Clinical data showed that iNOS elevated in endothelial cells, keratinocytes and renal tissue cells in SLE patients [22]. In animal models of SLE, increased NO and iNOS expression were observed [23]. Studies from both human and animals showed that iNOS over-expression has a positive association with autoimmune diseases [24]. iNOS inhibitor prevents glomerulonephritis developing in MRL/lpr mice [25, 26], which suggests that iNOS promote the development and progression of SLE. A recent study with MRL/lpr mice suggested iNOS promoted the proliferation of T follicular helper (Tfh) cells [27], which were thought to play a critical role in the pathogenesis of SLE though promoting B cells to produce more IgG [28, 29]. A positive correlation between iNOS and SLE had emerged. However, Bronte et al. indicated that iNOS can limit the functions of autoreactive T cells and reduce disease severity [30]. Meanwhile, a recent study by using NOS2^−/−^ MRL/lpr mice showed a lower level of anti-inflammatory cytokine IL-10 and a higher level of triglycerides, ceramide and sphingosine-1-phosphate (S1P) which aggravate vascular inflammatory processes, suggesting that iNOS may relieve SLE symptoms in some conditions [31]. Collectively, these data suggested a pathogenic role of iNOS in SLE. However, the role is controversial. Herein, we conducted this meta-analysis to explore the association between iNOS and SLE.

## Results

### Studies included

**Fig. 1:**
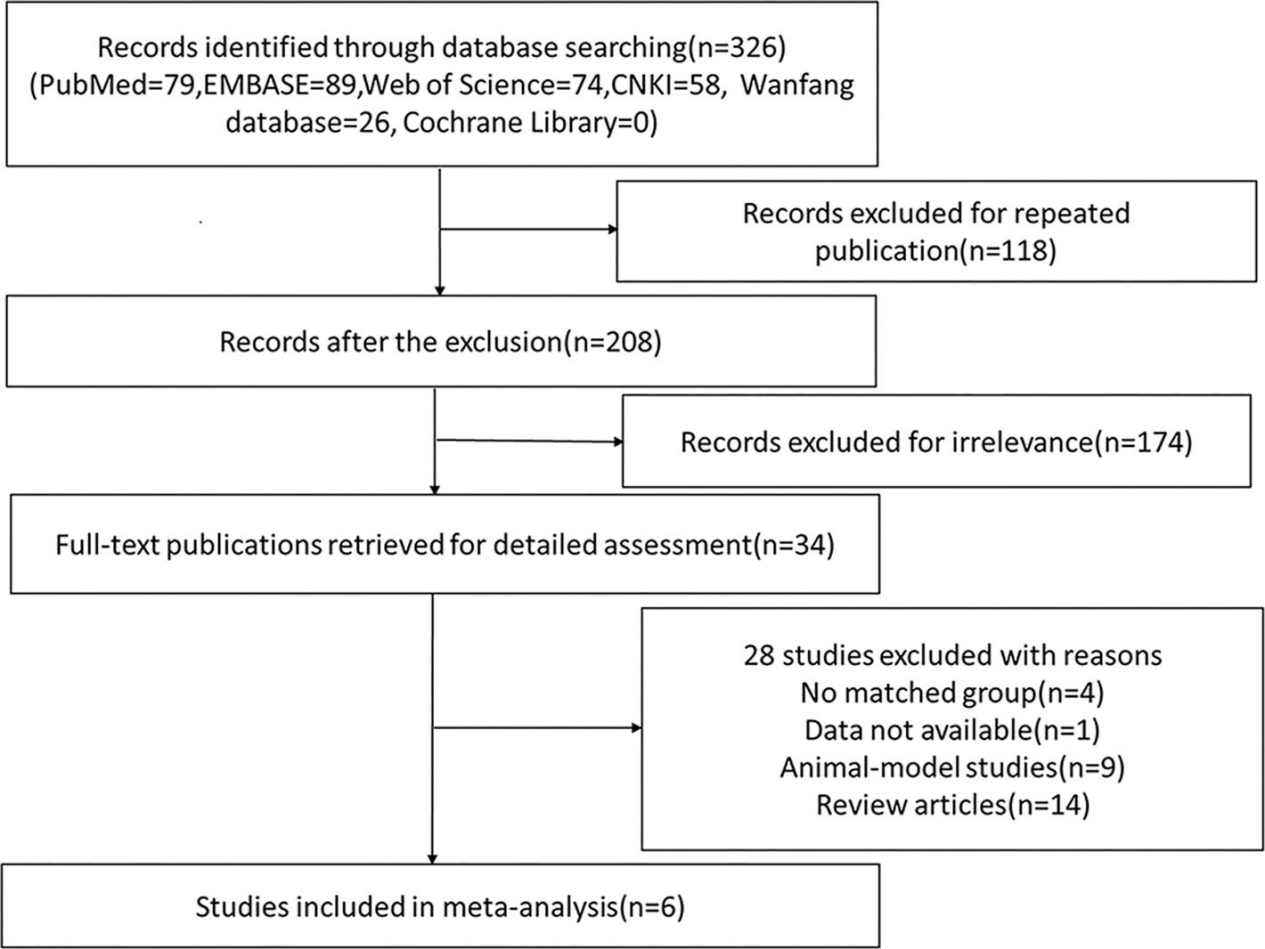
Flow diagram for identification of relevant studies

The detailed search process was showed in the flow diagram (Fig. 1). The search strategy retrieved 326 potentially relevant studies, which included 79 articles from PubMed, 89 from EMBASE, 74 from Web of Science, 58 from CNKI, 26 from Wanfang database, 0 from Cochrane Library. After excluding duplicate and irrelevant publications, 34 articles were scrutinized. According to the inclusion criteria, 6 articles were included in this meta-analysis finally [22, 32–36]. Total sample sizes of the 6 studies were 277, including 184 patients with SLE and 93 control subjects.

### Study characteristics

The major characteristics of the included studies were summarized in Table 1. All of them were designed as case-control studies. The age and gender were matched between SLE patients and control subjects in each included study. Of these, subjects of 3 studies [22, 33, 35] were fulfilled the criteria of the ARA for SLE diagnosis, the other 3 [32, 34, 36] were diagnosed according to ACR. The tissues used for evaluating iNOS expression were as follows: In studies of Belmont [32] and Kuhn [22] skin was evaluated, in studies of Gong [33] and Xu [34], peripheral blood was evaluated, and in the other 2 studies [35, 36] kidney was evaluated. The quality of included studies was assessed by Newcastle-Ottawa Scale. All studies showed high quality for achieving a rating of 5 stars or higher (see Table 2).

**Table 1:** Characteristics of individual studies included in the meta-analysis

Study	Study design	Sample size (SLE/control)	Gender of SLE(F/M)	Mean age of SLE(y)	Criteria of SLE diagnosis	Type of tissue/cell used in studies	Characteristics of controls
Belmont 1997 [32]	Case-control	36(25/11)	NG	NG	ACR	Skin: endothelial cell, keratinocyte	Skin biopsy samples obtained from young, healthy, female volunteers
Kuhn 1998 [22]	Case-control	26(21/5)	NG	NG	ARA	Skin: keratinocyte	Non-lesional skin from healthy people
Gong 2002 [33]	Case-control	64(34/30)	30/4	29.8	ARA	Peripheral blood: PBMC	Healthy volunteers, mean age was 25.6, gender ratio was 26/4
Xu 2003 [34]	Case-control	58(38/20)	38/0	32.2	ACR	Peripheral blood	Healthy volunteers, mean age was 28.2, gender ratio was 20/0
Zheng 2006 [35]	Case-control	59(49/10)	43/6	30.85	ARA	Kidney: tubular cell	Normal kidney biopsies without SLE, but accompanied by renal cell carcinoma
Bollain 2009 [36]	Case-control	34(17/17)	14/3	25.9	ACR	kidney	Normal kidney biopsies without renal pathology

*F* Female, *M* Male, *SLE* Systemic lupus erythematosus, *ARA* American Rheumatism Association, *ACR* American College of Rheumatology, *NG* Not given

**Table 2:** Study assessment using the Newcastle-Ottawa Scale

Study	Patient selection	Comparability	Exposure	Total stars
Belmont 1997 [32]	4*	1*	2*	7*
Kuhn 1998 [22]	3*	1*	1*	5*
Gong 2002 [33]	4*	1*	1*	6*
Xu 2003 [34]	3*	1*	2*	6*
Zheng 2006 [35]	4*	1*	1*	6*
Bollain 2009 [36]	4*	1*	2*	7*

* indicates item achieves 1piont in Newcastle-Ottawa Scale

### Expression of iNOS

**Fig. 2:**
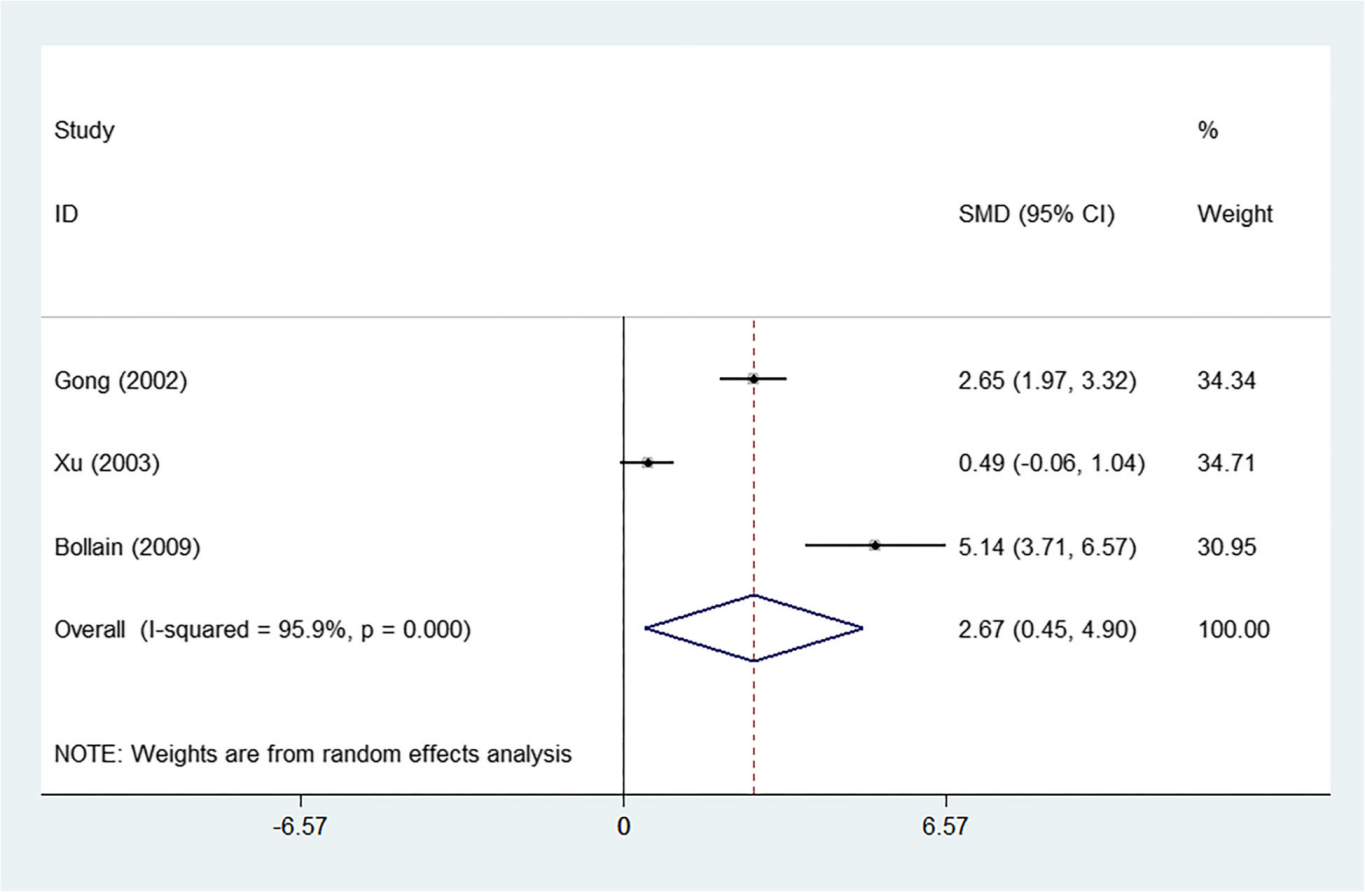
Forest-plot representing the expression of iNOS at mRNA level between SLE patients and controls

**Fig. 3:**
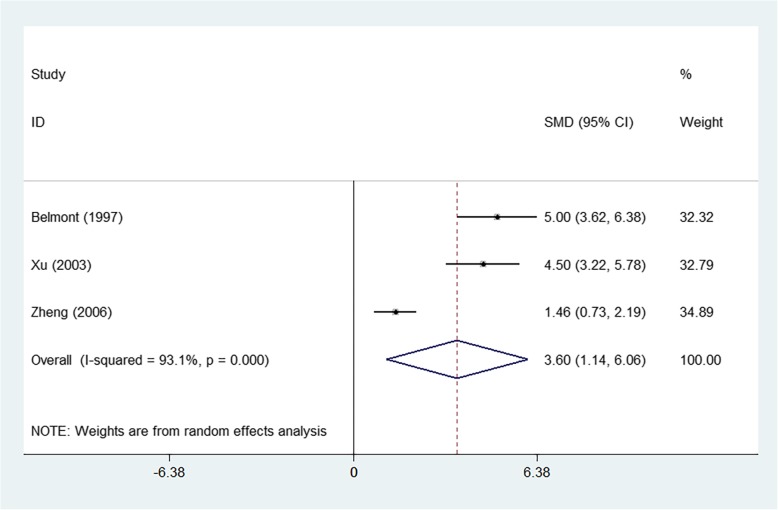
Forest-plot representing the expression of iNOS at protein level (staining score of iNOS) between SLE patients and controls

**Fig. 4:**
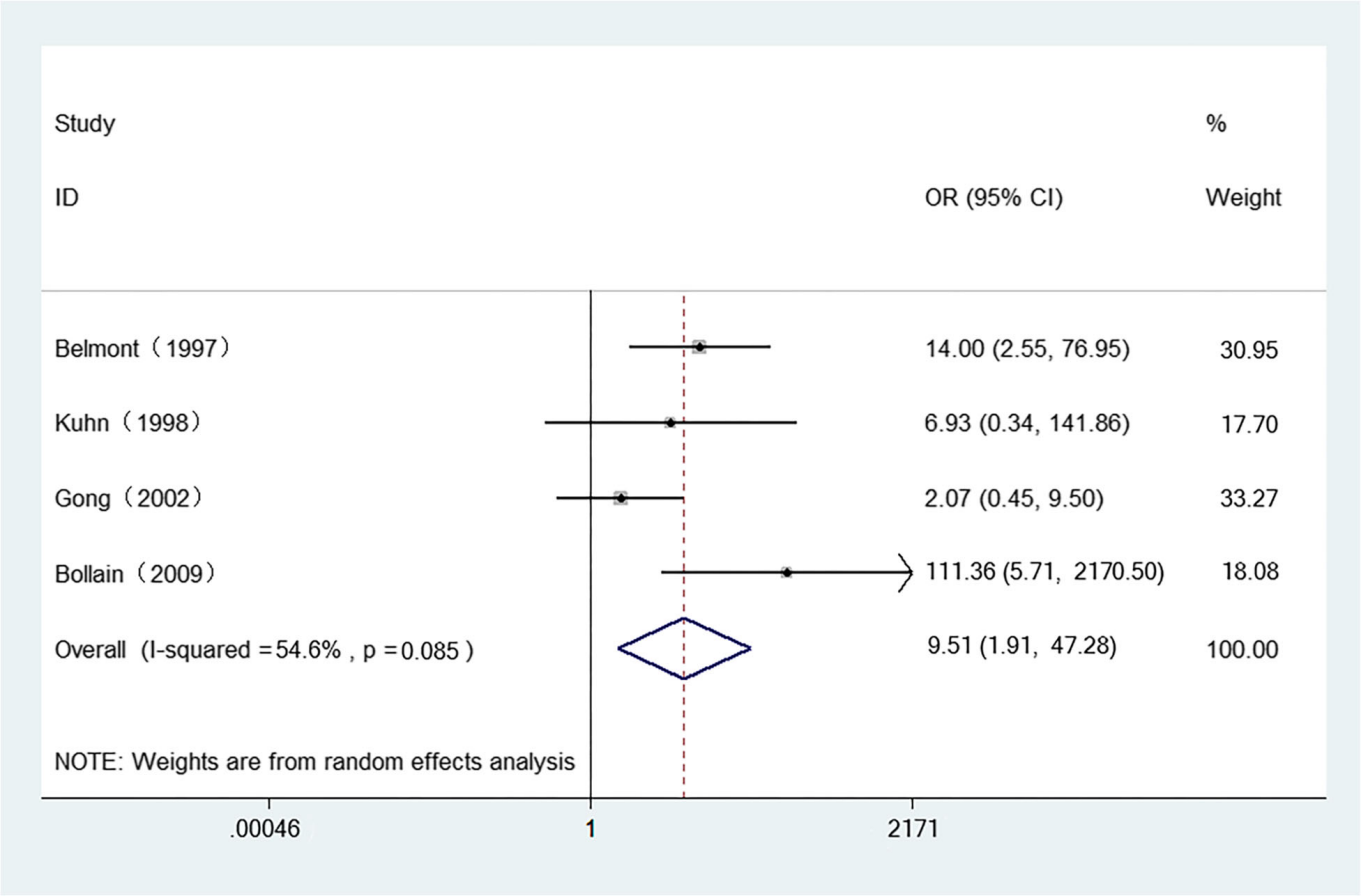
Forest-plot representing the positive rate of iNOS between SLE patients and controls

The meta-analysis showed that SLE patients had higher expression of iNOS at mRNA level than the controls (SMD = 2.671, 95%CI = 0.446–4.897, z = 2.35, *p* = 0.019) (Fig. 2 and Additional file 5: Figure S5A). And a similar tendency was observed at the protein level, staining score of iNOS (SMD = 3.602, 95%CI = 1.144–6.059, z = 2.87, *p* = 0.004) (Fig. 3 and Additional file 5: Figure S5B). Meanwhile, positive rate of iNOS (OR = 9.515, 95%CI = 1.915–47.281, z = 2.76, *p* = 0.006) (Fig. 4 and Additional file 5: Figure S5C) were significantly higher in SLE group compared with control group.

### Serum nitrite level

**Fig. 5:**
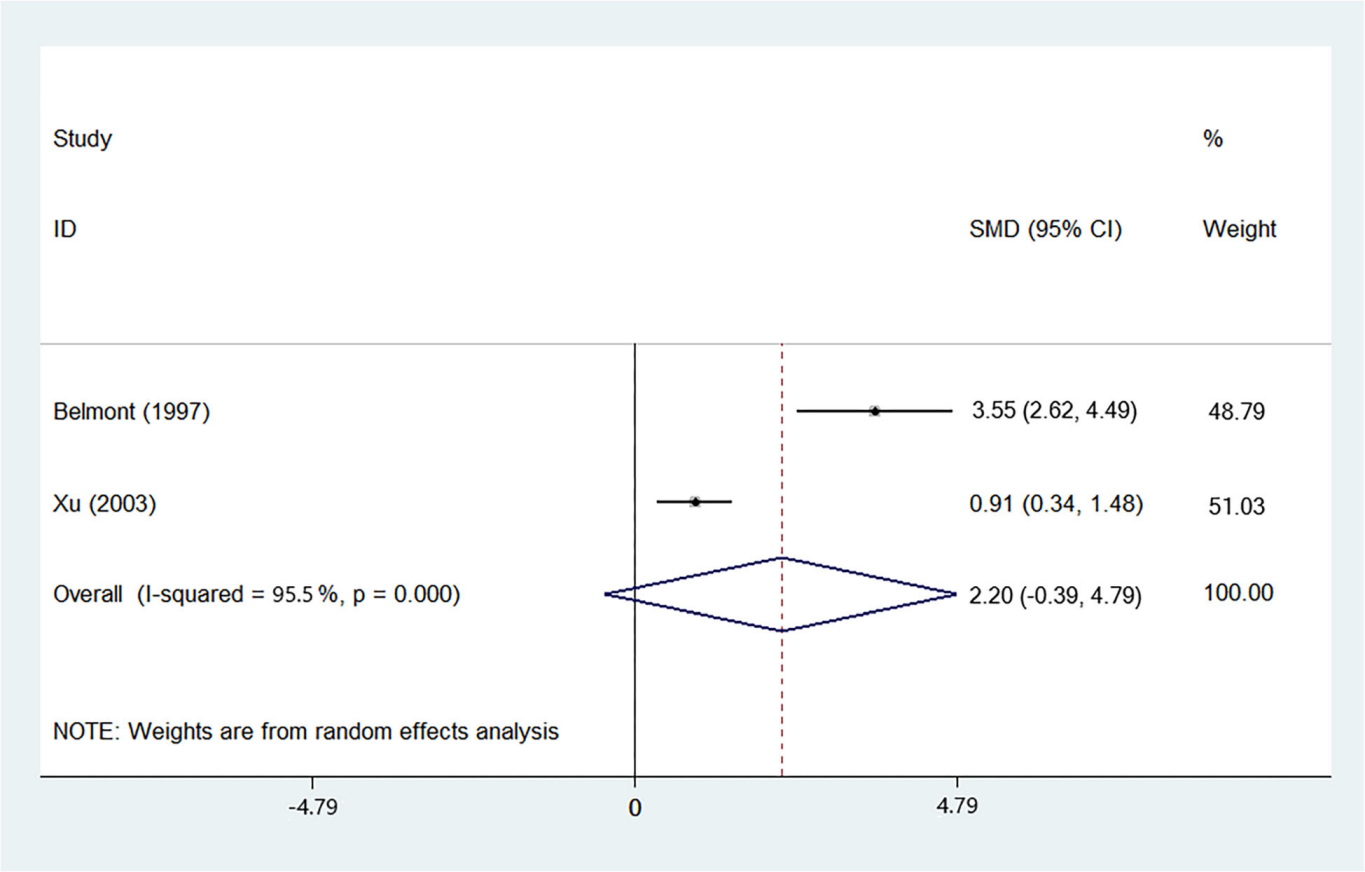
Forest-plot representing the serum nitrite elevation between SLE patients and controls

Serum NO is rapidly oxidized to nitrite by iNOS. Nitrite was measured as surrogate markers of NO. So, it always represents the level of NO. We did not observe significant differences in serum nitrite level between SLE cases and control subjects (SMD = 2.203, 95%CI = -0.386–4.793, z = 1.64, *p* = 0.095) (Fig. 5 and Additional file 5: Figure S5D).

### Assessment of publication bias

Because of the limits of funnel plotting, Egger’s test was performed to test publication bias*.* Except for iNOS expression at protein level (*p* = 0.006) there was no publication bias according to Egger’s test (Table 3). iNOS expression at mRNA level yieled an Egger’s test score of *p* = 0.326, Similar results were found in positive rate of iNOS (*p* = 0.337). However, it’s a failure to get the publication bias of serum nitrite because of insufficient studies.

**Table 3:** Egger’s test results of this meta-analysis

Publication bias	Studies(n)	Egger’s test	
		*P* value	95% CI
iNOS mRNA level	3	0.326	−64.87792, 85.96822
iNOS protein level	3	0.006	9.40983, 11.97152
Positive rate of iNOS	4	0.337	−177.3446, 322.8514

### Heterogeneity and sensitivity analysis

The results in this study showed significant level of substantial heterogeneity (I^2^ > 90%, *p* < 0.0001), except for the positive rate of iNOS (I^2^ = 54.6%, *p* = 0.085). Subgroup analysis on public year, sample size, study quality and assessing tissue type was tried, although the number of included studies was small. However, for the limitation of the insufficient studies we did not find the source of heterogeneity (Additional file 1: Figure S1 and Additional file 2: Figure S2).

Sensitivity analysis by excluding individual studies revealed that the results were not modified when compared to the overall effect (Additional file 3: Figure S3). Then the sensitivity analysis with fixed effects model demonstrated that the results were not changed (Additional file 4: Figure S4). Overall, sensitivity analysis revealed that the results produced in this meta-analysis were robust.

## Discussion

More and more experimental data suggest that iNOS was an essential pathogenic mediator in SLE. The general idea of this progress is that iNOS rises in SLE and the inhibitor of iNOS can lessen disease severity [24, 25]. However, several studies indirectly showed an opposite opinion that iNOS might relieve SLE symptoms [30, 31]. Our meta-analysis showed that the expression of iNOS is higher in SLE patients than control subjects, both at mRNA level and protein level (including the staining score and positive rate of iNOS expression). This is consistent with previous findings [5, 8, 37]. These data supported the hypothesis that over-expression of iNOS may lead to organ damage and alter immune response with SLE. Although the present analysis showed a positive correlation between iNOS and SLE, the source of iNOS is unknown. Recently, increasing evidence has suggested that proportions of myeloid-derived suppressor cells (MDSCs), a heterogeneous myeloid precursor, enhanced in SLE patients and mice [38, 39]. Although a variety of potential mechanisms is involved in the immunosuppressive function of MDSCs, the main suppressive activity of MDSCs is associated with the secretion of arginase-1 and iNOS [30, 40]. So, we speculate iNOS and MDSCs has some relationship in SLE. Perhaps MDSCs is the source of iNOS.

Previous studies show iNOS and excessive production of NO have been detected in SLE [12, 21]. NO can convert to nitrite, nitrate, nitrotyrosine (NT) and peroxynitrite (ONOO^−^), which are stable. So, nitrite is evaluated to represent NO level in general. Our analysis showed no significant difference in serum nitrite elevation between SLE group and control group. This goes against the previous studies [5, 12, 21]. It may be due to the small sample of this index. Besides, dual effect of NO may contribute to this result. Low levels of NO plays a role of defending factor against invading pathogens. High levels of NO plays a role of proinflammatory factor promoting immune disorders. However, there is no definite boundary of the concentration. Meanwhile, SLE patients are under the environment with inflammation for a long time, and if they have formed a tolerance of NO is not clear. Moreover, in arthritis and experimental autoimmune encephalomyelitis (EAE) models, NOS inhibition during inactive stage worsened clinical symptoms, while during active stage ameliorated clinical symptoms [41, 42]. The individuals included in the selected studies were accorded with ACR or ARA criteria of SLE diagnosis, but the included studies did not describe if they were during an active or inactive stage. All of this may infect the results.

iNOS is an inducible synthase and can catalyze arginase producing excessive NO. NO is one of the most important RNS. There is a wealth of evidence to implicate the increased RNS were associated with autoantibody increasing, suggesting a potential role of RNS in autoimmune disease, including SLE [43–45]. However, the exact mechanisms are not clear. According to the present information, we known NO has various proinflammatory actions that lead to tissue damage and chronic inflammation because of involving in several cell functions [46]. For example, NO can lead to gene mutation via the cytosine deamination, it also can lead to oxidative phosphorylation decreasing due to the mitochondrial iron-sulfur cluster enzyme inhibition [42]. ONOO^−^, one of the stable products of NO, is more reactive and more toxic than NO and permeates membranes to attack cellular compartments and nucleic acid in cells leading cell injury. It can induce apoptosis via cytochrome-C-mediated caspase activation. Circulating apoptotic cells can lead to accumulation of autoantibodies in SLE [47]. In addition, ONOO^−^ also may play a role in activating signaling pathways such as activator protein-1 (AP-1) and nuclear factor-kappa B (NF-κB). These studies together inspired the hypothesis that iNOS correlated with the progression of SLE via RNS. Previous studies have shown that JAK/STAT signaling pathway involves in the suppression of T cells by inducing iNOS [48]. Recently, some new views have been put forward. In macrophage, the induction of iNOS was dependent on PI3K/ AKT signaling pathways [49]. Umbilical cord mesenchymal stem cells (MSC) therapy lessened SLE mice by producing iNOS to inhibit T follicular helper (Tfh) cells expansion [27]. The study in murine animals shows L-NMMA, an iNOS inhibitor, can break the balance of immune tolerance in SLE via regulatory B (Breg) cells [50]. These findings suggest that iNOS can be considered as a novel targeting therapeutic strategy in SLE patients. Previous studies have shown that inhibiting iNOS activity in MRL/lpr mice can restore renal catalase activity, and inhibit cellular apoptosis and proliferation in the glomerulus [51, 52]. Also, heme-oxygenase-1 (HO-1), which can reduce the expression of iNOS, was an effective therapy for glomerulonephritis in MRL/lpr mice [53–55]. Although the mortality of SLE is greatly reduced, patients still suffer from disease flares due to the complex mechanisms. In line with this, immunosuppressant medications are mainstay of treatment. Several of the medications drive benefits from their capacity to inhibit iNOS. In some tissues, glucocorticoids and cyclosporine inhibit the induction of iNOS. Mycophenolate Mofetil can reduce the generation of NO through inhibiting the activity of GTP-dependent iNOS. These medicines showed effectiveness toward SLE. However, they can only prolong the inactive period of SLE instead of recovery. Despite the availability of new treatments directed against causative molecular targets, most clinical trials have been disappointing. Therefore, iNOS has important implications regarding the development of pharmacologic therapies for SLE.

The present study has some limits cannot be ignored. First, because of the limits of incorporated studies, the limited sample size may decrease the statistical power of this analysis. Second, all the included studies were case-control and single-center clinical studies, so they lacked randomness. Third, in the included criteria, we did not set a rigorous definition to the tissue which used to measure iNOS expression. For iNOS expression is positive in multiple cell types, such as macrophages, endothelial cells, peripheral blood monocytes, tubular cells [56, 57]. Moreover, there were no unified standards according to previous studies. This might be a confounding factor. Fourth, there was significant heterogeneity in our meta-analysis. The source of heterogeneity was unidentifiable despite attempting different sensitivity analyses and analyzing different subgroups. The insufficient number of included studies is the major limiting factor. Other factors, such as methods of measurement, sample size, public year, environmental factors, should also be taken into account. Finally, the result of iNOS expression at protein level has a publication bias, the source of it may come from 3 aspects: included studies were non-randomized controlled trails, the sample size was small, the high heterogeneity. Above all, the results should be interpreted with caution. More prospective randomized studies are needed to certify our results.

## Conclusions

This meta-analysis showed SLE patients have a higher iNOS expression at both mRNA level and protein level. On the contrary, there is no difference of serum nitrite level between SLE patients and control subjects. These data suggested that the over-expression of iNOS may relate to the pathological process of SLE. However, the source of iNOS is unclear. Although reactive nitrogen species are considered to be the most important and widely studied way to join in SLE, other factors are discovered gradually, such as Tfh [37], Breg cells [38], as well as some signaling pathways (PI3K/AKT and JAK/STAT signaling pathways). Therefore, therapeutic strategy targeting iNOS is a promising therapy for SLE patients.

## Methods

### Search strategy

The online database of PubMed, Web of Science, EMBASE, Cochrane Library, China National Knowledge Infrastructure (CNKI) and Wanfang database were searched for observational studies assessing the association of iNOS and SLE, Using the following key words or MeSH terms: “systemic lupus erythematosus” or “SLE” or “lupus”, combined with “inducible nitric oxide synthase” or “inducible NOS” or “iNOS” or “NOS_2_”. The final systematic search was conducted on June 2018 with no language restriction. References in the retrieved articles were explored for potentially relevant studies.

### Inclusion criteria

Articles were included if they met the following criteria:

1. Case-control, cross-sectional or cohort studies;
2. SLE patients were diagnosed based on the diagnostic criteria of American Rheumatism Association (ARA) or American College of Rheumatism (ACR);
3. Control subjects are people without the history of any autoimmune disorders including SLE;
4. Results contained evaluation of iNOS.

### Exclusion criteria

Articles were excluded if they met the following criteria:

1. animal-model studies, case reports, review articles, letters, comments, editorials;
2. Participants were pregnancy;
3. Participants who were suffering diabetes mellitus, malignant tumor, chronic kidney diseases and other diseases can lead to cachexia;
4. Studies with incomplete data.

### Data extraction

Data were extracted by 2 independent authors. Ambiguities were resolved by discussion among all authors. The third reviewer (Huanfa Yi) was the final person to solve the issue. Data from each study were extracted including, but not limited to: name of author, year of publication, study design, gender of participants, numbers of participants, age of participants, criteria of SLE diagnosis, tissue type used to evaluate iNOS.

### Quality assessment

As the studies involved in this meta-analysis are all case-control ones, quality of the included studies was assessed by Newcastle-Ottawa Scale (NOS). In this scale, “patient selection”, “comparability of study groups” and “exposure” consist of a particular “star system” to evaluate included studies [58]. The lowest score was 0 star, and the highest score was 9 stars. Studies with a score of ≥5 stars were defined as having a high quality. On the other hand, studies with a score of <5 stars were defined as having a low quality [59].

### Statistical analysis

Odd ratio (OR) was calculated with 95% confidence intervals (95% CI) for dichotomous variables. Standardized mean difference (SMD) was calculated with 95% confidence intervals (95% CI) for continuous variables. The Q statistic test and the I^2^ test were used to assess heterogeneity [60]. If I^2^ >50% (I^2^ test) and *p* <0.1 (Q test) were applied as significant heterogeneity of outcomes, we use random effect model to pool the data. Otherwise, the fixed effect model was used. The subgroup analysis was conducted to detect the sources of heterogeneity. The articles involved in our meta-analysis were less than 10, funnel plots cannot be used to detect publication bias [61]. Due to the limits of funnel plotting, publication bias was evaluated by Egger’s test, where, *p* < 0.05 was considered as statistically significant. We evaluated the strength of the pooled estimates by performing a leave-one-out sensitivity analysis and changing the analysis method from random-effects model to fixed-effects model to measure the impacts that each study applies on the overall pooled estimate. All statistical analysis involved in this meta-analysis was carried out with the use of the STATA 12.0. Statistically significant value was less or equal to 0.05 (*p* ≤ 0.05).

## Supplementary information

**Additional file 1: Figure S1.** Subgroup analysis of the expression of iNOS at mRNA level from A) Public year (1: ≤2002, 2: > 2002); B) Study quality (1: ≤6*, 2: > 6*); C) Sample size (1: > 50, 2:≤50); D) Tissue (1: skin, 2: blood, 3: kidney).

**Additional file 2: Figure S2.** Subgroup analysis of staining score of iNOS from A) Public year (1: ≤2002, 2: > 2002); B) Study quality (1: ≤6*, 2: > 6*); C) Sample size (1: > 50, 2:≤50); D) Tissue (1: skin, 2: blood, 3: kidney).

**Additional file 3: Figure S3.** Sensitivity analysis by excluding individual studies of different results: A) expression of iNOS at mRNA level; B) staining score of iNOS; C) positive rate of iNOS; D) serum nitrite level.

**Additional file 4: Figure S4.** Sensitivity analysis with fixed effects model of different results: A) expression of iNOS at mRNA level; B) staining score of iNOS; C) positive rate of iNOS; D) serum nitrite level.

**Additional file 5: Figure S5.** The statistical values (z value and *p* value) of combined effect variables of different results: A) expression of iNOS at mRNA level; B) staining score of iNOS; C) positive rate of iNOS; D) serum nitrite level.

## Data Availability

The datasets used and/or analyzed during the current study are available from the corresponding author on reasonable request.

## Abbreviations

3-NT: 3-nitrotyrosine
ACR: American College of Rheumatism
AP-1: Activator protein-1
ARA: American Rheumatism Association
CNKI: China National Knowledge Infrastructure
EAE: Experimental autoimmune encephalomyelitis
eNOS: Endothelial nitric oxide synthase
HO-1: heme-oxygenase-1
iNOS: Inducible nitric oxide synthase
MDSCs: Myeloid-derived suppressor cells
MSC: Mesenchymal stem cells
NF-κB: Nuclear factor-kappa B
nNOS: Neuronal nitric oxide synthase
NO: Nitric oxide
NOS: Nitric oxide synthase
NT: nitrotyrosine
ONOO^−^: Peroxynitrite
RNS: Reactive nitrogen species
S1P: Sphingosine-1-phosphate
SLE: Systemic lupus erythematosus
