# Supplementary figures and images for: Inducible nitric oxide synthase and systemic lupus erythematosus: a systematic review and meta-analysis
Source: BMC Immunol. 2020 Feb 17;21:6. doi: 10.1186/s12865-020-0335-7 (PMC7027241; doi:10.1186/s12865-020-0335-7)

A

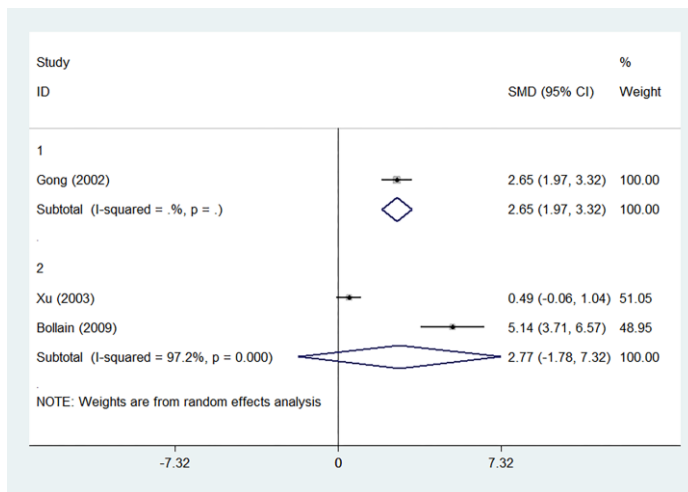

B

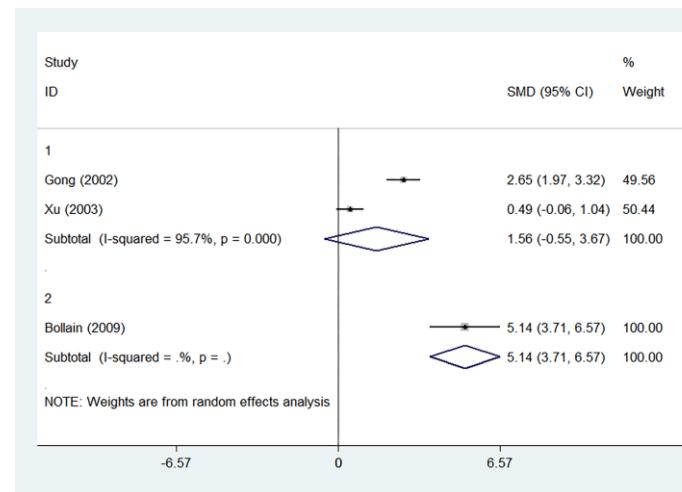

C

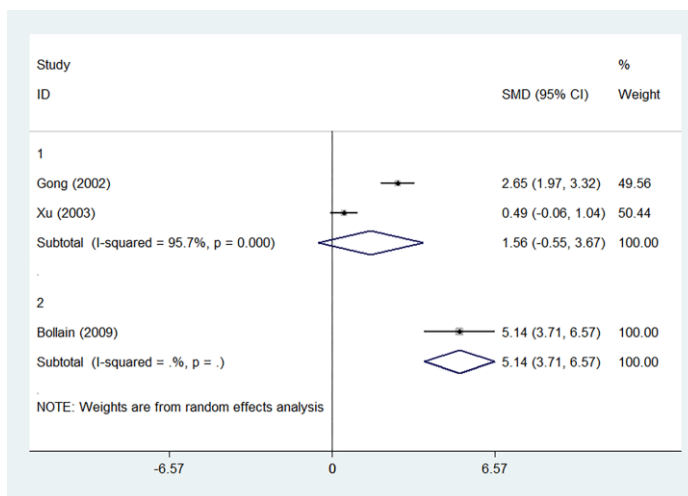

D

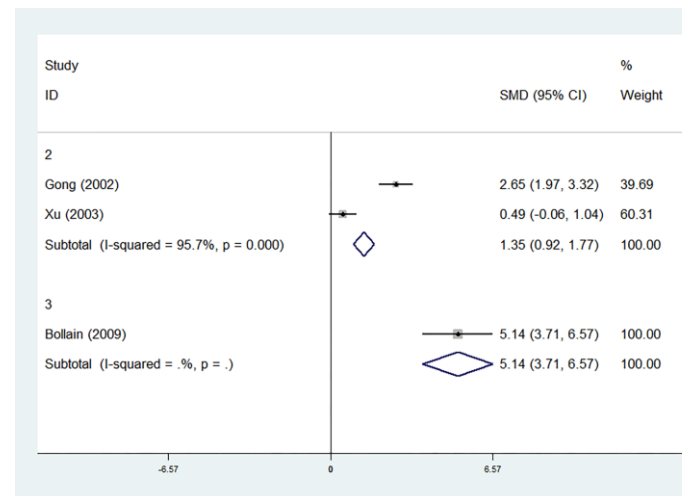

Supplement: Supplementary file 1 — Additional file 1: Figure S1. Subgroup analysis of the expression of iNOS at mRNA level from A) Public year (1: ≤2002, 2: > 2002); B) Study quality (1: ≤6*, 2: > 6*); C) Sample size (1: > 50, 2:≤50); D) Tissue (1: skin, 2: blood, 3: kidney). [file 12865_2020_335_MOESM1_ESM.pdf]

A

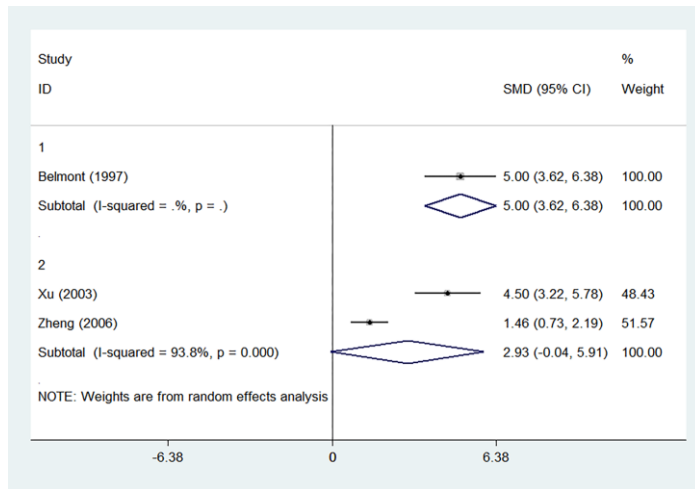

B

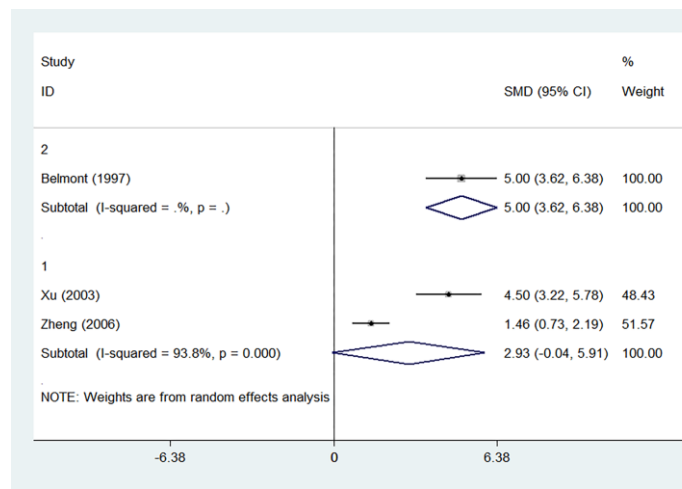

C

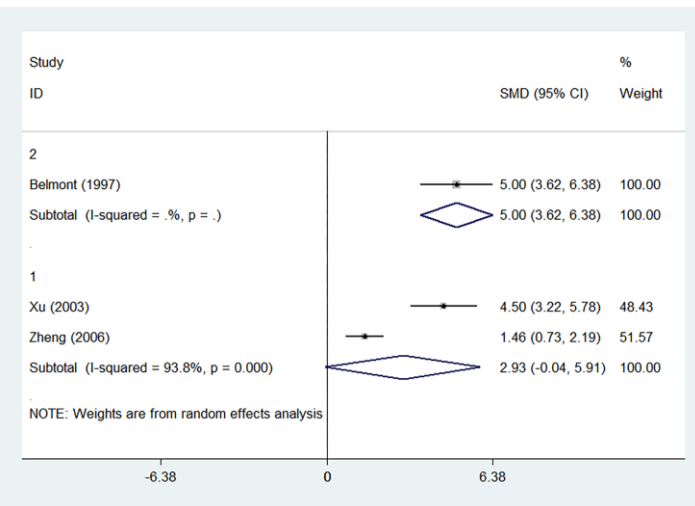

D

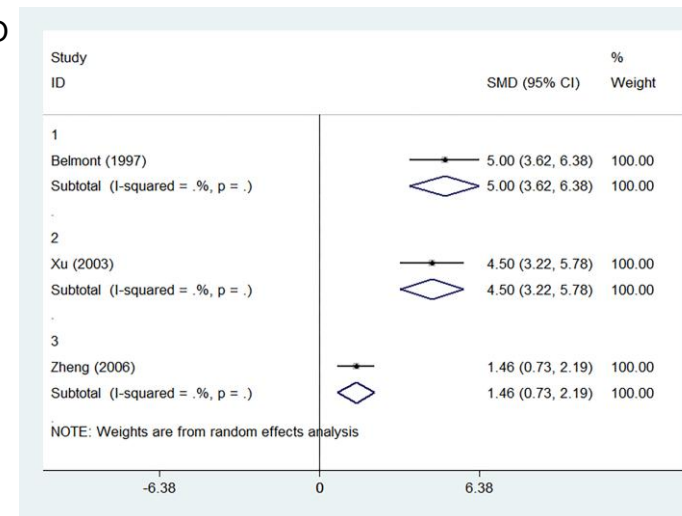

Supplement: Supplementary file 2 — Additional file 2: Figure S2. Subgroup analysis of staining score of iNOS from A) Public year (1: ≤2002, 2: > 2002); B) Study quality (1: ≤6*, 2: > 6*); C) Sample size (1: > 50, 2:≤50); D) Tissue (1: skin, 2: blood, 3: kidney). [file 12865_2020_335_MOESM2_ESM.pdf]

A

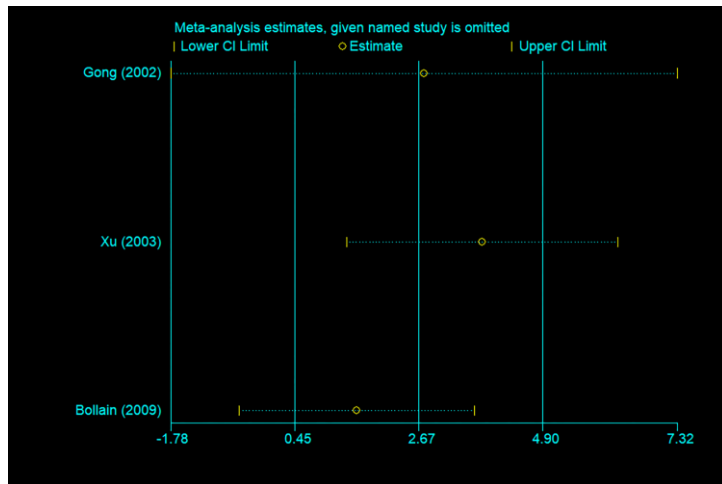

B

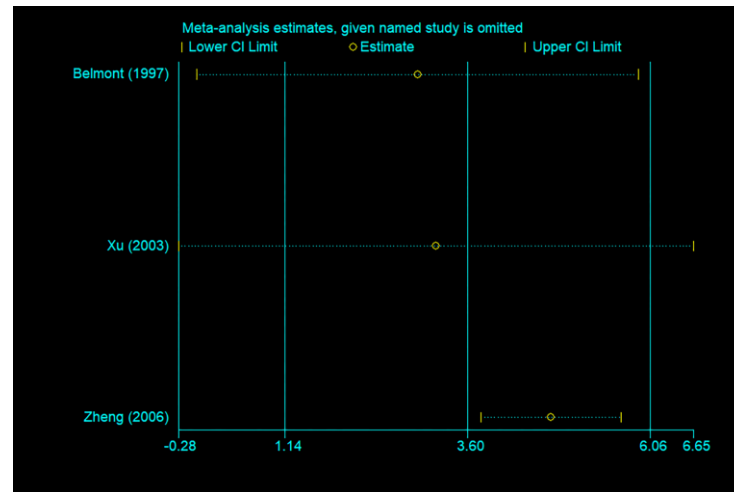

C

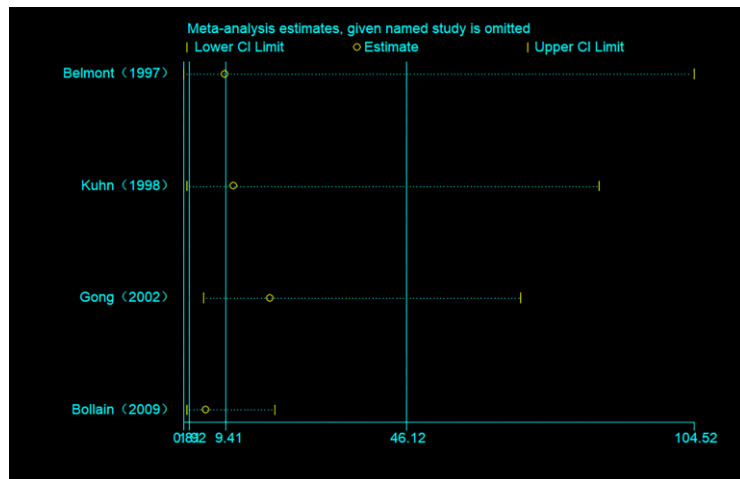

D

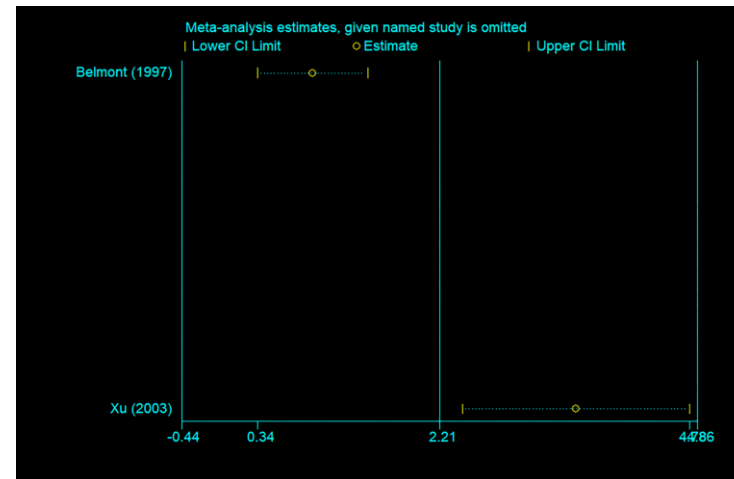

Supplement: Supplementary file 3 — Additional file 3: Figure S3. Sensitivity analysis by excluding individual studies of different results: A) expression of iNOS at mRNA level; B) staining score of iNOS; C) positive rate of iNOS; D) serum nitrite level. [file 12865_2020_335_MOESM3_ESM.pdf]

A

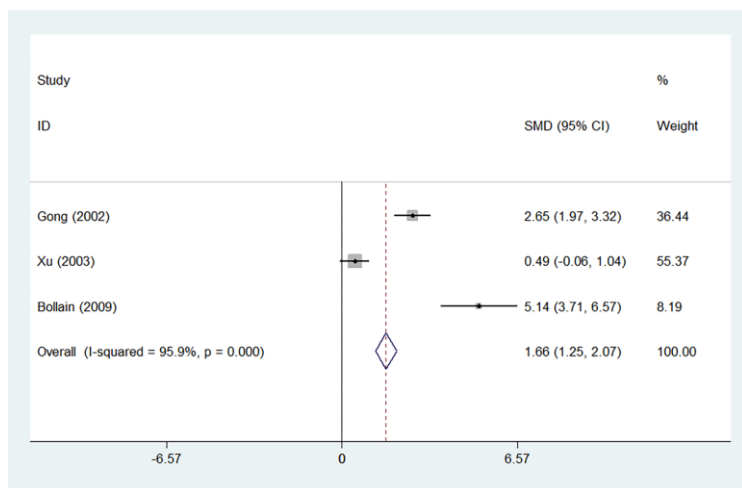

B

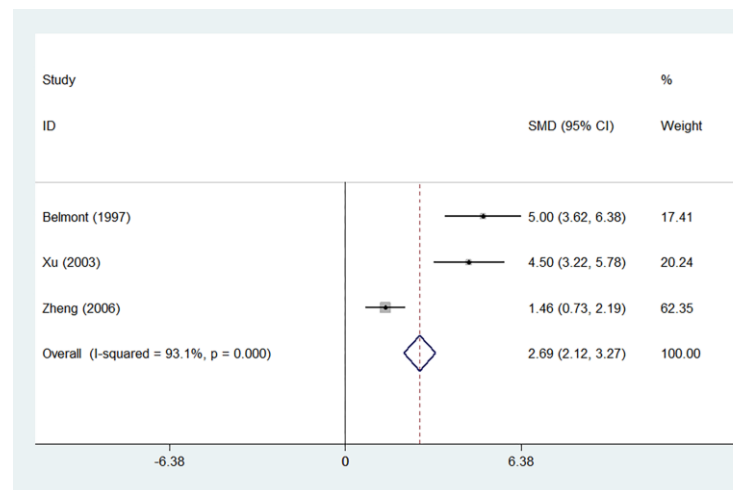

C

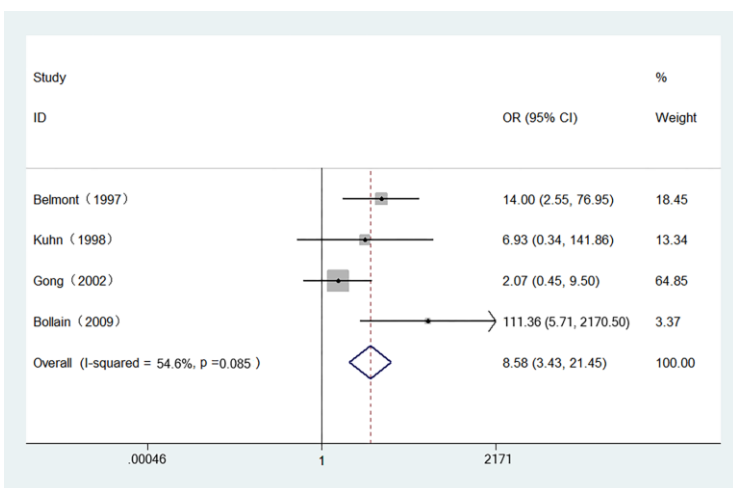

D

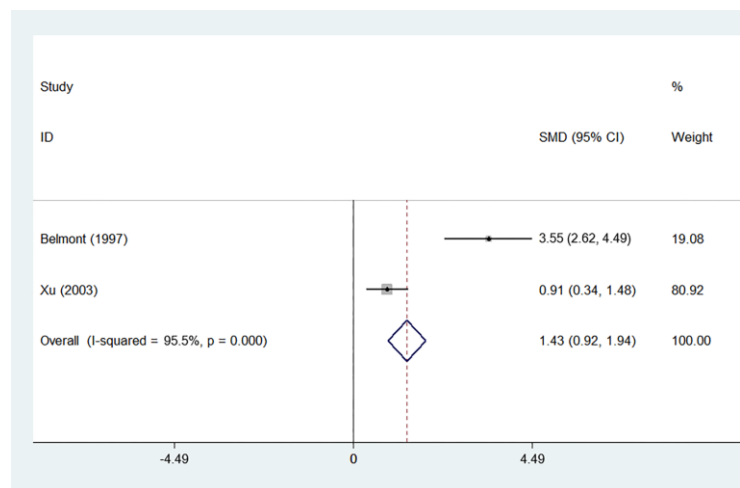

Supplement: Supplementary file 4 — Additional file 4: Figure S4. Sensitivity analysis with fixed effects model of different results: A) expression of iNOS at mRNA level; B) staining score of iNOS; C) positive rate of iNOS; D) serum nitrite level. [file 12865_2020_335_MOESM4_ESM.pdf]
