# Supplementary material for: Inducible nitric oxide synthase and systemic lupus erythematosus: a systematic review and meta-analysis
Source: BMC Immunol. 2020 Feb 17;21:6. doi: 10.1186/s12865-020-0335-7 (PMC7027241; doi:10.1186/s12865-020-0335-7)
Supplement: Supplementary file 5 — Additional file 5: Figure S5. The statistical values (z value and p value) of combined effect variables of different results: A) expression of iNOS at mRNA level; B) staining score of iNOS; C) positive rate of iNOS; D) serum nitrite level. [file 12865_2020_335_MOESM5_ESM.pdf]

A

Study	SMD	[95% Conf.	Interval]	% Weight
Gong (2002)	2.647	1.971	3.324	34.34
Xu (2003)	0.493	-0.056	1.043	34.71
Bollain (2009)	5.140	3.713	6.568	30.95
D+L pooled SMD	2.671	0.446	4.897	100.00

Heterogeneity chi-squared = 48.35 (d.f. = 2) p = 0.000
I-squared (variation in SMD attributable to heterogeneity) = 95.9%
Estimate of between-study variance Tau-squared = 3.6360

Test of SMD=0 : z= 2.35 p = 0.019

B

Study	SMD	[95% Conf.	Interval]	% Weight
Belmont (1997)	5.000	3.616	6.384	32.32
Xu (2003)	4.500	3.217	5.783	32.79
Zheng (2006)	1.463	0.732	2.194	34.89
D+L pooled SMD	3.602	1.144	6.059	100.00

Heterogeneity chi-squared = 29.17 (d.f. = 2) p = 0.000
I-squared (variation in SMD attributable to heterogeneity) = 93.1%
Estimate of between-study variance Tau-squared = 4.3662

Test of SMD=0 : z= 2.87 p = 0.004

C

Study	OR	[95% Conf.	Interval]	% Weight
Belmont (1997)	14.000	2.547	76.950	30.95
Kuhn (1998)	6.926	0.338	141.861	17.70
Gong (2002)	2.067	0.450	9.499	33.27
Bollain (2009)	111.36	5.710	2170.504	18.08
D+L pooled OR	9.515	1.915	47.281	100.00

Heterogeneity chi-squared = 6.47 (d.f. = 3) p = 0.085
I-squared (variation in OR attributable to heterogeneity) = 54.6%
Estimate of between-study variance Tau-squared = 1.3584

Test of OR=1 : z= 2.76 p = 0.006

D

Study	SMD	[95% Conf.	Interval]	% Weight
Belmont (1997)	3.552	2.621	4.492	48.97
Xu (2003)	0.909	0.342	1.478	51.03
D+L pooled SMD	2.203	-0.386	4.793	100.00

Heterogeneity chi-squared = 16.67 (d.f. = 1) p = 0.000
I-squared (variation in SMD attributable to heterogeneity) = 95.5%
Estimate of between-study variance Tau-squared = 3.4380

Test of SMD=0 : z= 1.64 p = 0.095
